# Vaccination a Relic of Barbarism

**Published:** 1882-02

**Authors:** 


### Vaccination a Relic of Barbarism.

Such is the heading of a diatribe against vaccination, in a journal before us professedly devoted to medical reform. Comment: In three of the local governments of India, the total deaths by small-pox in the year 1878 were 226,946. This was the happy result of comparative exemption from the "Relic of Barbarism." During the same year the number of deaths from small-pox in all Europe and America within the range of advanced civilization, was certainly not more than ten thousand—probably much less. So much for the "Relic of Barbarism." We advise our anti-vaccinationists to travel off to the Punjaub, where they may enjoy the fruits of exemption from the Jennerian curse. Civilization and humanity can well dispense with their reformatory service.—*Pacific Medical and Surgical Journal.*

